# Relevance and targeting of memory T cells in transplantation

**DOI:** 10.1186/ar3409

**Published:** 2011-09-16

**Authors:** Birgit Sawitzki, Anja Siepert, Manfred Lehmann, Undine Gerlach, Andreas Pascher, Stefan Tomiuk, Hans-Dieter Volk, Petra Reinke

**Affiliations:** 1 Institute of Medical Immunology, Charite Universitätsmedizin, Berlin, Germany; 2 Berlin Brandenburg Center for Regenerative Medicine, Berlin, Germany; 3 Institute of Medical Biochemistry and Molecular Biology, University of Rostock, Rostock, Germany; 4 Department of Visceral and Transplant Surgery, Charite Universitätsmedizin, Berlin, Germany; 5 Miltenyi Biotec GmbH, Bergisch-Gladbach, Germany; 6 Department of Nephrology and Internal Intensive Care Medicine, Charite Universitätsmedizin, Berlin, Germany

## 

Achieving tolerance or drug minimization after transplantation and thus preventing permanent immunosuppression with all the known severe side effects is the most important goal in transplantation medicine. In the last 20 years major progress has been made in understanding the tolerance underlying mechanisms and develop therapeutic strategies in small animal models. However, such knowledge could be rarely translated into the development of successful new therapeutic approaches in clinical transplantation. The success is limited by clinical challenges which are not present in our clean animal facilities such as 1) heterologous immunity - pathogen-specific memory T and B cells recognize alloantigens and boost the immune response towards the allograft, 2) pre-sensitization of recipients - presence of allo-specific memory T and B cells which are inert to most known therapeutic regimens. Thus we know now that we need more personalized treatment strategies according to the patient's immune reactivity. Such a strategy should combine three important aspects: i) an improved immune monitoring; ii) treatment which target memory cells and iii) strategies to reinforce regulation.

We have established preclinical transplant models with preformed allo-reactive or pathogen-specific memory T cells in which we compare effectiveness of different treatment approaches combining depletional with regulatory approaches.

Furthermore, we have performed a DNA microarray screen on samples of transplant patients and identified surface molecules specifically expressed by naïve, central memory, effector memory or terminal differentiated effector memory (TEMRA) T cells. Using this approach we hope to develop antibodies, which specifically deplete effector memory and TEMRA cells but spare naïve and central memory T cells. Such a treatment will be associated with less side effects e.g. infectious complications as compared to global depletion of T and B cells.

